# Comparison of serum lipid profile in Type-2 Diabetics with and without retinopathy in Pakistani population

**DOI:** 10.12669/pjms.326.11056

**Published:** 2016

**Authors:** Zulfiqar Ali Amin, Qamar Ul Islam, Mohammad Asim Mehboob

**Affiliations:** 1 Dr. Zulfiqar Ali Amin, FCPS (Med), FCPS (Medical Oncology). Department of Medicine, PNS Shifa Hospital, Karachi, Pakistan; 2 Dr. Qamar Ul Islam, FCPS (Ophthalmology), FCPS (VRO). Department of Ophthalmology, PNS Shifa Hospital, Karachi, Pakistan; 3 Dr. Mohammad Asim Mehboob, MBBS. Department of Ophthalmology, PNS Shifa Hospital, Karachi, Pakistan

**Keywords:** Lipid profile, Dyslipidaemia, Diabetic retinopathy, Diabetes mellitus

## Abstract

**Objective:**

To compare serum cholesterol, TG, HDL-C and LDL-C concentrations between type-2 Diabetes mellitus (DM) patients with retinopathy and without retinopathy and to study association between various modifiable risk factors of Diabetic retinopathy (DR).

**Methods:**

The study included 300 patients with type 2 DM; 140 of them were without DR (Group-I) and 160 were with DR (Group-II). Serum total cholesterol, LDL-C, HDL-C, and TG levels were determined. SPSS 17.0 for windows was used for statistical analysis

**Results:**

Overall, mean age of study population was 48.86 ± 5.62 years. Subjects with DR were older (*P* < 0.018), had higher fasting plasma glucose (*P* < 0.01) and higher HbA1c (*P* <0.01) concentrations compared with those without DR. Analysis of serum cholesterol, LDL-C, HDL-C and TG among subgroups of patients with no DR, with NPDR and PDR showed statistically significant difference (p <0.01). There was strong positive correlation of severity of DR with BSF, HbA1c, serum LDL-C, total cholesterol and TG.

**Conclusion:**

The serum cholesterol, TG, HDL-C and LDL-C concentrations were found to be significantly deranged in patients with DR as compared to those without DR.

## INTRODUCTION

Diabetes mellitus (DM) has been emerging as a major healthcare problem in Pakistan with 7.0 million people suffering from it and the number of diabetic patients is estimated to rise to a staggering figure of 14.4 million by the year 2040 making Pakistan the 8^th^ highest country in the world in terms of burden of diabetic patients.^1^ The morbidity and mortality related to DM is mainly attributed to its microvascular complications including retinopathy, nephropathy and neuropathy. Chronic hyperglycaemia, increased reactive oxygen species, decreased nitric oxides and increased fatty acids are responsible for these chronic vascular complications by altering the vascular response.^2^ The major ocular complication of DM is diabetic retinopathy (DR) which is the leading cause of irreversible blindness worldwide with prevalence of DR in newly diagnosed type II diabetics up to 40%.^3^,^4^ Known risk factors for development and progression of DR include type and duration, age, gender, body-mass index (BMI), glycaemic control, hypertension, nephropathy, smoking, pregnancy and serum lipid levels.^3^,^5^

Role of serum lipids in development and progression of DR has been evaluated worldwide with variable results. Diabetic dyslipidaemia characterized by elevated serum total cholesterol (TC), triglycerides (TG), low density lipoproteins cholesterol (LDL-C) and high density lipoproteins cholesterol (HDL-C) has been proposed as possible risk factors for DR.^6^,^7^ Hyperlipidaemia causes endothelial dysfunction due to reduced bioavailability of nitric oxide and breakdown of blood retinal barrier leading to exudation of serum lipids and lipoproteins which results in DR changes and diabetic macular odema (DME) formation.^8^,^9^ Data from Diabetes Control and Complications Trial (DCCT) showed serum lipids were positively associated with risk of DR in type I diabetics.^10^ Chennai Urban Rural Epidemiology Study (CURES), showed that the mean serum TC, TG and non HDL-C levels were higher in patients with DR as compared to those without DR.^11^ Cetin et al. in their study did not found significant association of serum lipid levels with the severity of DR.^9^ Ahsan et al. in their work on Pakistani population found significantly elevated serum cholesterol and serum creatinine levels in type II diabetic patients with sight threatening DR as compared to patients with no DR.^12^ Due to the variation in results from international studies and scarcity of data from local literature, this study was conducted to explore this intriguing relationship between serum lipids and DR in our population. The objective was to compare serum TC, TG, HDL-C and LDL-C concentrations between type- 2 DM patients with retinopathy and without retinopathy and to study association between various modifiable risk factors of DR.

## METHODS

This cross-sectional comparative study was conducted at the department of Medicine and Ophthalmology, PNS Shifa Naval hospital, Karachi from Jan 2016 to June 2016. After approval by the hospital ethical review committee, informed written consent was taken from the patients prior to inclusion in the study. Patients from both genders, aged between 35-70 years, with recent or earlier diagnosis of type 2 DM were included through non-probability convenience sampling. Patients with hypertension, heart disease, renal disease, use of anti-hyperlipidaemia drugs, and history of ophthalmic diseases or surgery were excluded. Calculated sample size was 140 in each group keeping level of significance as 0.5, power of test as 80, population mean of serum cholesterol value as 185.47 in DR group, and 169.31 in control group and population SD as 46.8.^7^

The pre devised proforma was completed by single researcher endorsing subject’s demography, and clinical profile. Fasting plasma glucose, serum TC, HDL-C, LDL-C and TG was measured by using Modular P- 800 (Roche Diagnostics, Manneheim, Germany) and HBA1C levels by Cobas C-111 (Roche Diagnostics, Manneheim, Germany) biochemistry machine after 8 hours of fasting. All patients were examined using slit lamp biomicroscopy by a single ophthalmologist after pupil dilation with 1% Tropicamide. Patients were assigned to two groups, based upon findings in worse eye. Group 1: no DR and Group 2: any type of DR. For detailed analysis DR group was further divided into patients with non proliferative DR (NPDR) and those with proliferative DR (PDR) on the basis of International Clinical Disease Severity Scale for DR.^13^ After performing all investigations, all patients were managed by physician and ophthalmologist according to their condition.

SPSS 17.0 for windows was used for statistical analysis. Descriptive statistics i.e. mean ± standard deviation for quantitative values (age, duration of DM, BMI, BSF, lipid sub fraction levels and HbA1C) and frequencies along with percentages for qualitative variables (gender, smoking status) were used to describe the data. Independent sample ‘t’ test and One way analysis of variance (ANOVA) with post hoc Tukey analysis was used to compare quantitative data between groups, while chi square test for independence was used to compare qualitative data. Pearson’s correlation coefficient test was performed to find association of different study variables. A p value < 0.05 was considered statistically significant.

## RESULTS

**Fig.1:**
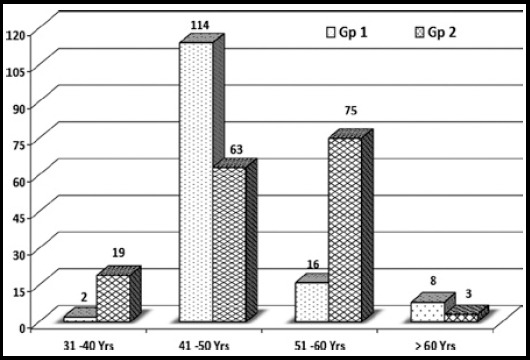
Group wise age spectrum.

Out of 300 subjects included in the study 160 had DR while 140 were without DR. Overall mean age of study population was 48.86 ± 5.62 years (range: 34-65 years) with 59% of the patients in their 5^th^ decades of life (Fig.1). GroupWise demographic and clinical data is shown in Table-I. Subjects with DR were older (*P* = 0.018), had higher fasting plasma glucose (*P* < 0.01) and higher HbA1c (P < 0.01) concentrations compared with those without DR. However, both the groups were matched in terms of gender (p=0.285), BMI (p=0.418) and duration of DM (p=0.067). Smoking tendency as a risk factor for DR was found to significantly higher in patients with DR (p< 0.01).

**Table-I:** Clinical and biochemical profile of study population.

Variable	Group 1 No DR (n = 140)	Group 2 With DR (n = 160)	P value
Age (years)	48.04 ± 4.83	49.58 ± 6.16	0.018
Male, n (%)	71 (50.71%)	91 (56.87%)	0.285
Smoker, n (%)	32 (22.85%)	67 (41.87%)	< 0.01
Duration (years)	4.60 ± 3.03	5.20 ± 2.61	0.067
BMI (kg/m^2^)	26.31 ± 2.71	26.59 ± 3.18	0.418
Plasma Glucose (F) mg/dl	117.34 ± 7.93	131.10 ± 14.27	< 0.01
HbA1C (%)	5.77 ± 0.50	7.71 ± 1.02	< 0.01

Analysis of serum levels of TC, LDL-C, HDL-C and TG among subgroups of patients with no DR, with NPDR and PDR showed statistically significant difference (p < 0.01) (Table-II). On post hoc analysis difference in serum HDL-C levels between NPDR and PDR groups was not statistically significant (p= 0.598). There was strong positive correlation of severity of DR with fasting plasma glucose (r=0.602, p <0.01), HbA1c (r=0.853, p <0.01), LDL-C (r= 0.764, p <0.01), TC (r= 0.799, p <0.01) and TG (r =0.772, p <0.01), whereas, age (r = 0.331, p <0.01) and duration of DM (r = 0.287, p <0.01) showed moderately positive correlation with severity of DR. Smoking (r = - 0.195, p = 0.001) and HDL-C levels (r = - 0.303, p <0.01) showed moderate inverse correlation with severity of DR. Correlation of DR with gender or BMI was not statistically significant. Similarly strong positive correlation was found between serum TC, TG, LDL-C with both fasting plasma glucose and HbA1c, while HDL-C showed weak negative correlation with both BSF and HbA1c.

**Table-II:** Lipid sub fraction values among subgroups.

Lipid Profile	No DR (n=140)	NPDR (n=109)	PDR (n=51)	P value
Serum Cholesterol (mg/dl)	187.26 ± 17.46	216.39 ± 7.53	234.06 ± 10.31	< 0.01
Serum LDL-C (mg/dl)	92.59 ± 11.53	122.62 ± 20.65	138.27 ± 4.68	< 0.01
Serum HDL-C (mg/dl)	45.63 ± 4.44	43.36 ± 2.77	42.75 ± 3.35	< 0.01
Serum TG (mg/dl)	169.28 ± 9.83	220.61 ± 34.06	240.98 ± 17.38	< 0.01

## DISCUSSION

Reported prevalence of DM in Pakistani population varies between 15% – 56.9%.^4^,^12^,^14^-^16^ DR is a major complication of DM, with sight-threatening disease present in approximately 10% of persons with diabetes.^17^ The association of hyperlipidemia or dyslipidemia with DR is still not well established. It has been postulated that an increase in blood viscosity and alterations in the fibrinolytic system, incorporation of triglycerides into the cell membrane and endothelial dysfunction occur in hyperlipidemia and lead to the formation of hard exudates, haemorrhage and odema in the retina.^11^ In our study, analysis of serum lipid sub fractions among subgroups of patients with no DR, with NPDR and PDR showed statistically significant difference (p <0.001). In Wisconsin Epidemiologic Study of Diabetic Retinopathy (WESDR) serum TC was not a significant factor in the severity of retinopathy but was significantly associated with the presence (odds ratio (OR) 1.65) and severity of hard exudates in subjects with young-onset DM.^18^ Javadi et al. in a population-based survey in Tehran reported that DM and hyperlipidemia was not significantly associated with the presence of DR in logistic regression analyses.^5^ By contrast, the Singapore Malay Eye Study recently reported that higher BMI and higher serum TC and LDL-C levels were associated with a lower prevalence of DR.^19^ However, various other studies showed significantly deranged lipid profile in patients with DR as compared to healthy controls or patients without DR. Ahsan et al in their study on Pakistani population reported that patients with sight threatening DR had significantly higher serum cholesterol levels as compared to patients with no DR.^12^ Rahman et al found that TG was significantly higher and HDL-C was significantly lower in patients with NPDR as compared to patients without DR.^6^ In studies on Indian population, Gnaneswaran et al.^7^ found significantly higher values of serum TC and LDL-C levels in DR patients, whereas, Rema et al.^11^ reported significantly higher mean serum TC, TG and non- HDL-C concentrations in subjects with DR. In studies comparing patients with DR and age matched healthy non diabetic controls, Kiran et al.^2^ reported significantly deranged lipid profile in DR patients, while Agarwal et al.^20^ found a statistically significant lower value of mean HDL-C in diabetic patients with or without DR. Rimpal P et al found serum TG, LDL-C and TC were elevated and serum HDL-C was decreased in diabetic subjects as compared to healthy control, whereas serum TG, TC and LDL –C were more elevated in those with retinopathy than that those without retinopathy.^21^

We found strong positive correlation between severity of DR with BSF, HbA1c, serum LDL-C, TC and TG, whereas, age and duration of DM showed moderately positive correlation with severity of DR. Smoking and serum HDL-C levels showed moderate inverse correlation with severity of DR. Correlation between DR with gender or BMI was not statistically significant. Ahsan et al in their study reported male gender (3.5 times), increased duration of diabetes (≥10 years, 5.46 times) and poor glycemic control (HbA1c ≥7%, 1.39 times) as significant factors for developing retinopathy.^12^ Agroivi et al. documented significant positive correlation of severity of DR with smoking, duration of DM, serum LDL-C and TG, whereas, strong inverse correlation was found with serum HDL-C.^8^ In CURES, subjects with DR were older, had longer duration of diabetes, lower BMI, higher plasma glucose and higher HbA1c concentrations compared with those without DR.^11^ They also found that severity of retinopathy significantly increased with increase in age and duration of diabetes and prevalence of all grades of DR was higher among men.^11^ In our study, strong positive correlation was found between serum TC, TG, LDL-C with both BSF and HbA1c, while HDL-C showed weak negative correlation with both BSF and HbA1c. In another study by Cetin et al, mean plasma glucose correlated significantly with serum TC and LDL-C while, mean HbA1c correlated significantly with serum TC and TG.^9^ Prakash et al. in their work reported that patients with severe DR had longer duration of diabetes and higher A1C, but they found no association between DR severity and blood sugar levels.^22^

Wide variation in the findings of different studies may be attributed to different ethnicity, dietary habit, life style, heterogeneity in subject selection, differences in the methodology of assessment and sample size. Based on the findings of this study, it is recommended that stringent measures must be adopted to control modifiable risk factors associated with development and progression of DR in order to reduce the morbidity related with this disease.

## CONCLUSION

Serum cholesterol, LDL-C and TG levels were significantly elevated and serum HDL-C level was decreased in patients with DR indicating a need to promptly address these modifiable risk factors in order to reduce the morbidity related to DR.
